# UQCRFS1N assembles mitochondrial respiratory complex-III into an asymmetric 21-subunit dimer

**DOI:** 10.1007/s13238-018-0515-x

**Published:** 2018-03-06

**Authors:** Shuai Zong, Jinke Gu, Tianya Liu, Runyu Guo, Meng Wu, Maojun Yang

**Affiliations:** 1 Ministry of Education Key Laboratory of Protein Science, Tsinghua-Peking Joint Center for Life Sciences, Beijing Advanced Innovation Center for Structural Biology, School of Life Sciences, Tsinghua University, Beijing, 100084 China; 2 School of Pharmacy, Tongji Medical College, Huazhong University of Science and Technology, Wuhan, 430030 China

## Dear Editor,

Mitochondrial respiratory chain consists of four multimeric protein complexes, Complex I-IV (CI, NADH dehydrogenase; CII, succinate:ubiquinone oxidoreductase; CIII, cytochrome *bc*_1_ complex; and CIV, cytochrome c oxidase). These four complexes transfer electrons from NADH or FADH_2_ to oxygen and pump protons from mitochondrial matrix to intermembrane space, generating electrochemical gradient across the inner membrane which is harnessed by complex V to synthesize ATP, providing the majority of energy acquired by living organisms. Respiratory chain complexes were reported to interact with each other to form supercomplexes, even megacomplex (Guo et al., 2017). However, despite decades of intensive research, many intriguing new findings concerning respiratory chain still emerging frequently in recent years (Baradaran et al., 2013; Guo et al., 2016; Schagger and Pfeiffer, 2000; Vinothkumar et al., 2014). CIII is the middle part of respiratory chain, transferring electrons from ubiquinone to cytochrome c, and pumping protons in the meantime. All previous studies reported that CIII from vertebrate species was a homodimer consisting of 22 subunits in total (Fernandez-Vizarra and Zeviani, 2015, 2017), while after analysis of previous structural data, we draw a different conclusion.

**Figure 1:**
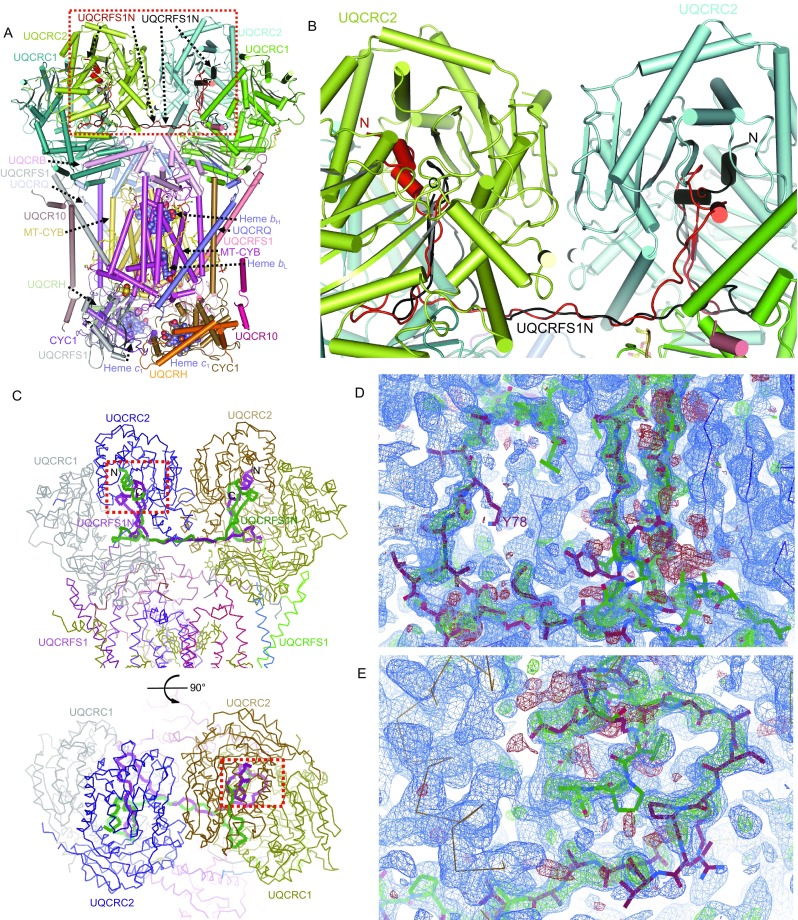

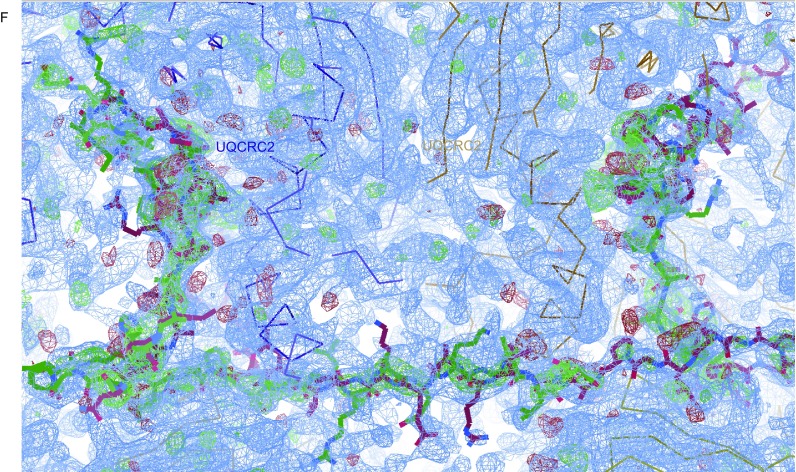
**UQCRFS1N assembles the 21-subunit CIII**. (A) The overall refined structure model of the 21-subunit asymmetric bovine CIII (PDB: 2A06). Different subunits of CIII are labelled and shown in different colors. The two possible orientations of UQCRFS1N are shown in red and black, respectively. (B) Structure of the UQCRFS1N subunit in two possible orientations. N and C termini of UQCRFS1N are labelled. This panel is the details of the red dashed frame in (A). (C) The UQCRFS1N structures of bovine CIII (PDB: 2A06). The left panel is viewed along the inner membrane, while the right panel is viewed from the matrix side. UQCRFS1N subunits of two possible orientations are shown in green and purple, respectively. Different subunits of CIII are labeled and shown in different colors. N terminal and C terminal of UQCRFS1N are labeled. The red dashed frames indicate two different binding regions of UQCRFS1N in UQCRC2 and UQCRC1. This figure is generated in COOT. (D) The density map of the region indicated by the left red dashed frame in (C). The UQCRFS1N subunits in two switched orientations are shown in sticks and in different colors. Green and blue meshes are mFo-DFc and 2mFo-DFc, respectively. This figure is generated by COOT. (E) The density map of the region indicated by the right red dashed frame in (C, bottom panel). Shown in the same way as (C, bottom panel). (F) The UQCRFS1N maps of chicken CIII (PDB: 3TGU). Shown in the same way as (C)

The structures of the CIII homodimer from different species have been extensively studied, with the first structure of bovine CIII solved in 1997 by the Deisenhofer group (Xia et al., 1997). Since then, a total of 46 (23 bovine, 17 chicken, 2 porcine, 3 ovine, and 1 human) CIII structures from vertebrate species are available till now (Fernandez-Vizarra and Zeviani, 2017). In all these structures, CIII was descripted as a homodimer. However, unlike other subunits, the UQCRFS1N subunit in all these structures is poorly defined. Although several structures have been solved with very high resolution, the full length N-terminal processed peptide (1–78 amino acids, UQCRFS1N) of the iron-sulfur Rieske protein (UQCRFS1) subunit has not been assigned in all of these structures (Table 1). UQCRFS1N is the N-terminal mitochondrial targeting sequence of UQCRFS1, and after its cleavage from the precursor, this small peptide remains bound to CIII with unknown functions. In this letter, we show that one UQCRFS1N links the two 10-subunit CIII protomers together to form the intact CIII, which resultantly contains only 21 subunits rather than previously assumed 22 subunits (Fig. 1A and 1B).

**Table 1:** Modelled UQCRFS1N residues in published CIII structures

Species	PDB ID	Method	Modelled UQCRFS1N residues	Resolution (Å)
*Bos taurus*	1BE3	X-ray	46–78	3.0
*Bos taurus*	1BGY	X-ray	46–78	3.0
*Bos taurus*	1L0L	X-ray	1–57	2.35
*Bos taurus*	1L0N	X-ray	1–57	2.6
*Bos taurus*	1NTK	X-ray	1–57	2.6
*Bos taurus*	1NTM	X-ray	1–57	2.4
*Bos taurus*	1NTZ	X-ray	1–57	2.6
*Bos taurus*	1NU1	X-ray	1–57	3.2
*Bos taurus*	1PP9	X-ray	32–78	2.1
*Bos taurus*	1PPJ	X-ray	32–78	2.1
*Bos taurus*	1QCR	X-ray	21–48	2.7
*Bos taurus*	1SQB	X-ray	1–57	2.69
*Bos taurus*	1SQP	X-ray	1–57	2.7
*Bos taurus*	1SQQ	X-ray	1–57	3.0
*Bos taurus*	1SQV	X-ray	1–57	2.85
*Bos taurus*	1SQX	X-ray	1–57	2.6
*Bos taurus*	2A06	X-ray	32–78	2.1
*Bos taurus*	2FYU	X-ray	1–57	2.26
*Bos taurus*	4D6T	X-ray	50–58, 66–77, 62–78	3.57
*Bos taurus*	4D6U	X-ray	50–63, 66–78, 62–78	4.09
*Bos taurus*	5KLV	X-ray	20–29, 46–64, 66–70	2.65
*Bos taurus*	5LUF	Electron microscopy	46–78	9.1
*Bos taurus*	5NMI	X-ray	49–78	3.5
*Gallus gallus*	1BCC	X-ray	-	3.16
*Gallus gallus*	2BCC	X-ray	-	3.5
*Gallus gallus*	3BCC	X-ray	-	3.7
*Gallus gallus*	3CWB	X-ray	47–77	3.51
*Gallus gallus*	3H1H	X-ray	47–77	3.16
*Gallus gallus*	3H1I	X-ray	47–77	3.53
*Gallus gallus*	3H1J	X-ray	47–77	3.0
*Gallus gallus*	3H1K	X-ray	47–77	3.48
*Gallus gallus*	3H1L	X-ray	47–77	3.21
*Gallus gallus*	3L70	X-ray	47–77	2.75
*Gallus gallus*	3L71	X-ray	47–77	2.84
*Gallus gallus*	3L72	X-ray	47–77	3.06
*Gallus gallus*	3L73	X-ray	47–77	3.04
*Gallus gallus*	3L74	X-ray	47–77	2.76
*Gallus gallus*	3L75	X-ray	47–77	2.79
*Gallus gallus*	3TGU	X-ray	2–8, 46–75	2.7
*Gallus gallus*	4U3F	X-ray	48–77	3.23
*Sus scrofa*	5GPN	Electron microscopy	46–78	5.4
*Sus scrofa*	5GUP	Electron microscopy	1–57	4.0
*Ovis aries*	5J4Z	Electron microscopy	1–57	5.8
*Ovis aries*	5J7Y	Electron microscopy	1–57	6.7
*Ovis aries*	5J8K	Electron microscopy	1–57	7.8
*Homo sapiens*	5XTE	Electron microscopy	1–57	3.4
*Homo sapiens*	5XTE	Electron microscopy	1–57	3.4

Firstly, we rebuilt the high-resolution crystal structures of bovine CIII (PDB: 2A06) (Huang et al., 2005) and chicken CIII (PDB:3TGU) (Hao et al., 2012). To do this, we downloaded the bovine structure (PDB: 2A06), the chicken structure (PDB:3TGU) and their corresponding MTZ files. We deleted UQCRFS1N residues in those two models and refined them with their maps thus generating two meshes named mFo–DFc and 2mFo–DFc, colored with green and blue, respectively. Analysis was processed after modelling of UQCRFS1N back into the corresponding densities. Intriguingly, the 2.1 Å density map of bovine CIII clearly shows that it contains two opposite orientations of the full-length UQCRFS1N peptide (1–78 amino acids), with the N terminal binding in one CIII protomer and the C terminal binding in the other CIII protomer (Fig. 1C–E). Both orientations can fit very well with the refined map, where the terminals of the two orientations occupy different densities while the middle regions of the two orientations share the same density. Refinement of the highest resolution chicken structure (PDB:3TGU) (Hao et al., 2012) gave same results (Fig. 1F).

This composition is conserved in vertebrates. Unconventionally, we propose that a single UQCRFS1N molecule tethers the two 10-subuint CIII protomers, with the N-terminal segment (residues 1–36) bound to one protomer and the C-terminal segment (residues 42–78) bound to the other. Furthermore, from the extra densities, we could build another UQCRFS1N molecule into CIII in a switched orientation, with the N- and C-terminal segments of the peptide inserting into the opposite CIII protomers (Fig. 1A and 1B). The two UQCRFS1N molecules partially overlap with each other, indicating that they could not simultaneously exist in the same complex. Thus, they likely represent alternative conformations of CIII. Thus, each CIII complex has 21 subunits. Twenty subunits (each protomer containing ten subunits) form a symmetric dimer while the UQCRFS1N peptide inserts into the two protomers with two alternative orientations to assemble them together (Fig. 1A and 1B).

The unusual binding mode of the UQCRFS1N peptide in CIII has not been previously observed. We checked 29 mammalian CIII (23 from bovine, 2 from porcine, 3 from ovine and 1 from human) and 17 chicken CIII structures available in the RCSB PDB protein data bank (https://www.rcsb.org), and found that none of them contains the full-length UQCRFS1N peptide (Table 1), consistent with a previous report (Fernandez-Vizarra and Zeviani, 2017). All previous structural studies on CIII suggested that this complex was a symmetric homodimer. However, our refinement of bovine and chicken structures (PDB: 2A06 and 3TGU) indicates that a single UQCRFS1N peptide can insert into the two CIII protomers with two different orientations when CIII is assembled into the respirasome (Fig. 1A and 1B). This further suggests that CIII has no preferred orientation when it binds to CI and CIV to form the respirasome (Gu et al., 2016; Wu et al., 2016). Re-refinement of the previous high-resolution X-ray bovine and chicken CIII structures (PDB: 2A06 and 3TGU) suggests that a single UQCRFS1N peptide in two opposite orientations can tether the two CIII protomers together (Fig. 1A and 1B), even in the absence of CI and CIV. This phenomenon is further consistent with our recent findings that both protomers of CIII have the abilities to bind with CI to form the megacomplex-I_2_III_2_IV_2_ (Guo et al., 2017).

The full length UQCRFS1 is the last subunit to be incorporated into CIII. Previous studies suggested that the tetra-tricopeptide repeat domain 19 (TTC19) protein collaborates with an inner membrane protease SLP2-PARL-YME1L (SPY) complex to remove UQCRFS1N (residues 1–78 amino acids) from the full length UQCRFS1 after its incorporation into CIII (Bottani et al., 2017; Wai et al., 2016). In the mitochondria of the *Ttc19*^*-/-*^ knockout mouse, the native molecular mass of CIII was slightly higher than that of CIII isolated from the wild type animals. This small difference might be the incorporation of two UQCRFS1N segments (which are still parts of the full-length UQCRFS1) into CIII, as opposed to the one UQCRFS1N subunit in the mature CIII. More recently, it was suggested that the CIII core subunits, UQCRC1 and UQCRC2, have the matrix processing peptidase (MPP) activity to cleave the UQCRFS1N peptide after UQCRFS1 is incorporated into the nascent pre-CIII dimer (Fernandez-Vizarra and Zeviani, 2017). On the other hand, UQCRFS1N could inhibit the MPP activity of the UQCRC1-UQCRC2 complex *in vitro* (Deng et al., 2001). Our structures show that one UQCRFS1N molecule can bind to both cavities formed by UQCRC1 and UQCRC2 in both protomers. It is unclear whether UQCRC1-UQCRC2 is indeed involved in the processing of UQCRFS1. More biological experiments are needed to clarify how UQCRFS1 is processed and how CIII is assembled.

In conclusion, after refinement of previous high resolution crystal structures of CIII, we find UQCRFS1N can incorporate into CIII dimer in two possible orientations, indicating CIII is a 21-subunit asymmetric dimer rather than a 22-subunit homodimer, and both orientations of UQCRFS1N can exist in respirasome, supporting that both protomers of CIII in respirasome are functional and are possible to interact with CI to form the megacomplex I_2_III_2_IV_2_.
